# Real-World Outcomes in Transplant-Ineligible Patients With Newly Diagnosed Multiple Myeloma Treated With Bortezomib/Cyclophosphamide/Dexamethasone and Bortezomib/Lenalidomide/Dexamethasone as Upfront Treatment

**DOI:** 10.7759/cureus.58999

**Published:** 2024-04-25

**Authors:** Nabiha Saeed, Zurrya Khan, Hamzah Jehanzeb, Taha Shaikh, Usman Shaikh, Salman N Adil, Varisha Madni, Hania Fatima, Umm E Abiha, Natasha Ali

**Affiliations:** 1 Department of Oncology, Aga Khan University Hospital, Karachi, PAK; 2 Department of Oncology, Aga Khan University Medical College, Karachi, PAK; 3 Department of Pathology and Laboratory Medicine/Oncology, Aga Khan University Hospital, Karachi, PAK

**Keywords:** bortezomib, transplant ineligible, proteasome inhibitors, triplet regimen, newly diagnosed multiple myeloma

## Abstract

Introduction

Multiple myeloma (MM) is a hematological disorder characterized by aberrant multiplication of malignant plasma cells in the bone marrow. The current mainstay of treatment for patients with newly diagnosed MM (NDMM) is a triplet regimen with a proteasome inhibitor, immunomodulatory imide, and dexamethasone. The two most common of these triplet regimens are VLD (bortezomib/lenalidomide/dexamethasone) and VCD (bortezomib/cyclophosphamide/dexamethasone). This study aims to compare the outcomes between these two therapies in transplant-ineligible patients with NDMM.

Methods

We conducted a retrospective study at the Aga Khan University Hospital in Karachi, Pakistan. All NDMM transplant-ineligible patients either receiving VLD or VCD therapy between January 2015 and December 2022 were included in our study. Hematological parameters before and after treatment were obtained from hospital records. Response to treatment was classified according to the International Myeloma Working Group (IMWG) response criteria as either complete response (CR), very good partial response (VGPR), partial response (PR), minimal response (MR), stable disease (SD), or progressive disease (PD). The response to treatment as well as overall survival (OS) and progression-free survival (PFS) was compared between VCD and VLD therapy. A p-value of 0.05 or less was taken to be statistically significant.

Results

Twenty (23.8%) patients in the VCD group and 20 (23.0%) in the VLD group underwent complete remission. Seven (8.3%) patients experienced disease progression in the VCD group, while the figure stood at three (3.4%) in the VLD group. There was no statistically significant difference in the overall response rate between the VCD (58; 69.0%) and VLD (70; 80.5%) groups (p=0.086), a difference that was not statistically significant on the Chi-square test. OS was comparable between VCD (69.1 months, 95%CI: 61.3-77.0) and VLD (76.9 months, 95%CI: 69.0-85.0) therapies.

Conclusions

The study did not identify any statistically significant distinction in the treatment outcomes between the VCD and VLD regimens among NDMM patients ineligible for transplantation. Nevertheless, the study highlights the positive outcomes observed with both treatments in this specific patient cohort. This implies that either regimen could be deemed suitable as a treatment option for patients in low- and middle-income countries. Since both regimens demonstrate comparable effectiveness, assessing the cost-effectiveness of these regimens is crucial. Future research should also explore the economic aspects of the two treatment options.

## Introduction

Multiple myeloma (MM) is a hematological disorder characterized by aberrant multiplication of malignant plasma cells in the bone marrow. Malignant plasma cell infiltration into bone and other tissues causes a wide variety of symptoms, which include anemia, hypercalcemia, renal insufficiency, osteolytic bone lesions, and impaired immune function. MM accounts for 1% of all malignancies and approximately 10% of all hematological malignancies [1]. It is more frequently found in males than in females, and African Americans have a prevalence rate of two times that of Caucasians [2]. The median age of diagnosis is 70 years [3]. 

Virtually all instances of MM develop from an asymptomatic premalignant illness called monoclonal gammopathy of undetermined significance (MGUS) [4]. In around 1% of cases each year, MGUS develops into MM or a comparable malignancy [5]. In certain patients with MM, the asymptomatic but more advanced pre-malignant stage known as smoldering MM (SMM) may be detected clinically [6]. SMM develops into MM at a rate of roughly 10% per year during the first five years after diagnosis, 3% per year during the following five years, and 1.5% per year beyond that [7]. 

When one or more myeloma-defining events (MDE) are present, together with the presence of 10% or more clonal plasma cells in the bone marrow or a biopsy-proven plasmacytoma, a diagnosis of MM is made [1]. MDEs include CRAB (calcium elevation (hypercalcemia), renal failure, anemia, or bone abnormalities (lytic bone lesions)) attributable to the plasma cell disorder, bone marrow clonal plasmacytosis ≥60%, serum involved/uninvolved free light chain (FLC) ratio ≥100 (provided involved FLC is ≥100 mg/L), or >1 focal lesion on magnetic resonance imaging [1]. 

Better survival, tolerability, and deeper response have been achieved by combining novel and immunomodulatory agents into the MM treatment paradigm. Augmented survival and long-term outcomes have been observed with the use of immunomodulatory agents and proteasome inhibitors combination [8,9]. A triplet regimen with a proteasome inhibitor, immunomodulatory imide, and dexamethasone has become a keystone of induction therapy for newly diagnosed MM (NDMM) [10]. Bortezomib and dexamethasone-based triplet regimens in combination with cyclophosphamide and lenalidomide are the two most frequently used induction regimens in NDMM.

Our study is a retrospective cohort study conducted at the Aga Khan University Hospital in Karachi, Pakistan, aiming to compare the outcomes of transplant-ineligible patients with NDMM undergoing VLD (bortezomib/lenalidomide/dexamethasone) and VCD (bortezomib/cyclophosphamide /dexamethasone) therapy as their primary treatment modality.

## Materials and methods

Study population and setting

This was an observational retrospective study conducted at the Aga Khan University Hospital in Karachi Pakistan, spanning patients from January 2015 to December 2022. All adult patients (over the age of 18) with transplant-ineligible NDMM treated with a combination of upfront VCD or VLD outside of clinical trials were included in the study. Patients with transplant-eligible NDMM, relapsed/refractory MM, and those with plasma cell leukemia, solitary plasmacytoma, or amyloidosis were excluded.

The study received approval from the Ethical Review Committee of Aga Khan University (approval number: 2023-8740-24859) and the College of Physicians and Surgeons of Pakistan (CPSP). Data collection was conducted through the electronic medical records of eligible patients, ensuring patient confidentiality. Information pertaining to demographics, response criteria for myeloma, and clinical outcomes were recorded in a standardized proforma.

Clinico-haematological parameters and treatment schedule

All patients underwent pre-chemotherapy assessments including physical examination, routine hematology workup including bone marrow biopsy, biochemistry testing including serum protein electrophoresis (SPEP), free light chain (FLC) ratio, immunofixation (IFE), and immunoglobulin levels along with Revised International Staging System (R-ISS) risk stratification before commencement of therapy. Patients with deranged renal function tests and a higher risk of thromboembolism received VCD while others received VLD therapy, irrespective of their R-ISS risk stage. Both VCD and VLD therapies were given a four-week cycle. In VCD therapy, bortezomib 1.3 mg/m^2^ was given either subcutaneously or intravenously, and cyclophosphamide 300 mg/m^2^ and dexamethasone 40 mg/m^2^ were both given orally (each given on days 1, 8, 15, and 22). In VLD therapy, bortezomib and dexamethasone were given as in the VCD regimen while lenalidomide 25 mg was given orally for 21 days during each cycle.

Response to therapy

Response to treatment was assessed after four cycles with protein electrophoresis, immunofixation, and bone marrow biopsy. Patients thereafter were monitored with close follow-up every one to two months. Response to the primary line of therapy was classified according to the International Myeloma Working Group (IMWG) criteria as either being a complete response (CR), very good partial response (VGPR), partial response (PR), minimal response (MR), stable disease (SD), or progressive disease (PD) [11]. These were defined as follows: (i) CR was defined as a negative serum and urine immunofixation with no evidence of soft tissue plasmacytoma and bone marrow aspirate demonstrating less than 5% clonal plasma cell, (ii) VGPR was taken to be a serum and urine M protein detectable by immunofixation but not on electrophoresis or at least a 90% reduction in serum M protein with a urine M protein <100 mg/24 hours, (iii) PR was defined as a  ≥50% reduction in serum M protein and reduction of 24-hour urinary M protein by 90% or to <200 mg/24 hours, (iv) MR was defined as ≥25% but ≤49% reduction of serum M protein and reduction in 24-hour urine M protein by 50-89%. If present at baseline, a ≥50% reduction in the size of soft tissue plasmacytoma is also required for MR, (v) SD was defined as any condition not meeting the criteria for CR, VGPR, PR, or PD, and (v) PD was defined as any of the following: a serum M protein increase ≥1 g/dL denotes PD, an absolute increase in Urine M protein of ≥200 mg/24 hours, a bone marrow plasma cell percentage with absolute increase must be ≥10%, or a ≥50% increase in the size or development of new bone lesions or soft tissue plasmacytoma. Stringent complete response (sCR) was not evaluated due to relative unavailability and high cost of FLC ratio.

Additionally, the overall response rate was defined as an outcome equivalent to a partial response or better, and progression-free survival (PFS) was defined as the duration between initiation of systemic treatment and disease progression. Overall survival (OS) was defined as the duration between the date of diagnosis and death, or censored at the date of last follow-up.

Statistical analysis

Basic demographic data and clinico-hematological parameters were summarized as percentage frequencies if categorical, and as means with their standard deviations if continuous. The percentage of patients falling in each therapy response category after initial systemic therapy was summarized as a percentage of patients for whom data on the response was available. A Kaplan-Meier survival analysis was conducted to determine and compare the mean survival times and PFS for patients undergoing VLD and VCD therapy. 

A chi-square test was also used to compare the overall response rate between these two groups. Additionally, a Cox regression was conducted, taking basic demographic variables, clinico-hematological parameters, and the first line of therapy as moderating variables. A p-value of 0.05 or less was taken to be statistically significant. All analyses were conducted using IBM SPSS Statistics for Windows, Version 26.0 (Released 2019; IBM Corp., Armonk, New York, United States).

## Results

Our study consisted of 180 patients of MM with a mean age of 62.1 years (SD=10.8 years). The sample was unevenly split between males (61.1%, n= 110) and females (38.9%, n= 70). Of these 180 patients, 38.3% (n=69) suffered from hypertension, 29.4% (n=53) had diabetes mellitus, and 10.6% (n=19) had ischemic heart disease. Baseline hematological parameters have been summarized in Table 1. At the time of diagnosis, the most common findings on serum immunofixation were IgG kappa monoclonal (44.4%, n=80), IgG lambda monoclonal (22.8%, n=41), and IgA kappa monoclonal (8.9%, n=16). Additionally, only 1.1% (n=2) patients were positive for Tp53 deletion. Patients mostly presented with stage II R-ISS disease (42.8%, n=77), followed by stage III (35.6%, n=64), and stage I (21.7%, n=39). All patients presented with bone lytic lesions. The sample was equally split between the group receiving VCD and the group receiving VLD, with 90 patients in each group (50.0%, n=90). The mean number of chemotherapy cycles received was 7.6 (SD=4.0).

**Table 1:** Baseline demographic and clinical characteristics of the study population Categorical variables have been represented as n (number of individuals in the category) and % (percentage of the overall sample represented by the category) in the form: n (%). Continuous variables have been represented as the mean value in our sample along with SD (standard deviation) in the form: mean ± SD. LDH: lactate dehydrogenase; R-ISS: Revised International Staging System

Variable	n (%)/mean ± SD
Gender	
Male	110 (61.1)
Female	70 (38.9)
Age (years)	62.1±10.8
Laboratory parameters at diagnosis	
Percentage of plasma cells (%)	41.6 ± 23.7
Free light chain ratio	89.4 ± 287
Serum creatinine levels (mg/dL)	2.2 ± 2.4
Serum calcium levels (mg/dL)	9.36 ± 1.682
Serum LDH levels (I.U/L)	285.03 ± 329.967
Beta-2 microglobulin levels (ng/mL)	12620.2 ± 15381.3
Albumin g/dL	3.3 ± 0.6
Tp53 Deletion	2 (1.1)
Serum Immunofixation	
IgA Kappa Monoclonal	16 (8.9)
IgA Lambda Monoclonal	14 (7.8)
IgG Kappa Monoclonal	80 (44.4)
IgG Lambda Monoclonal	41 (22.8)
Kappa Light Chain	11 (6.1)
Others	18 (10)
R-ISS	
Stage I	39 (21.7)
Stage II	77 (42.8)
Stage III	64 (35.6)
Primary Chemotherapy Regimen	
VCD	90 (50.0)
VLD	90 (50.0)
Number of Chemotherapy Cycles Received	7.6 ± 3.9

**Figure 1:**
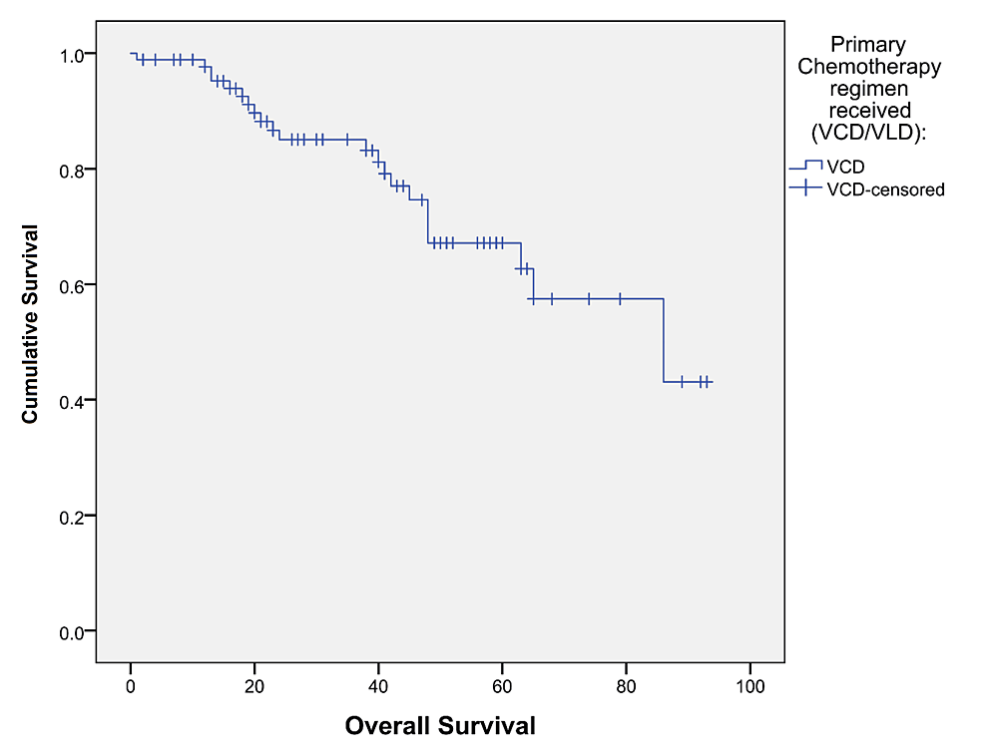
Kaplan-Meier survival graph of the group receiving VCD as the primary chemotherapy regimen The X-axis represents the overall survival in months, while the Y-axis represents the survival function represented as the cumulative survival. VCD: bortezomib/cyclophosphamide /dexamethasone; VLD: bortezomib/lenalidomide/dexamethasone

**Figure 2:**
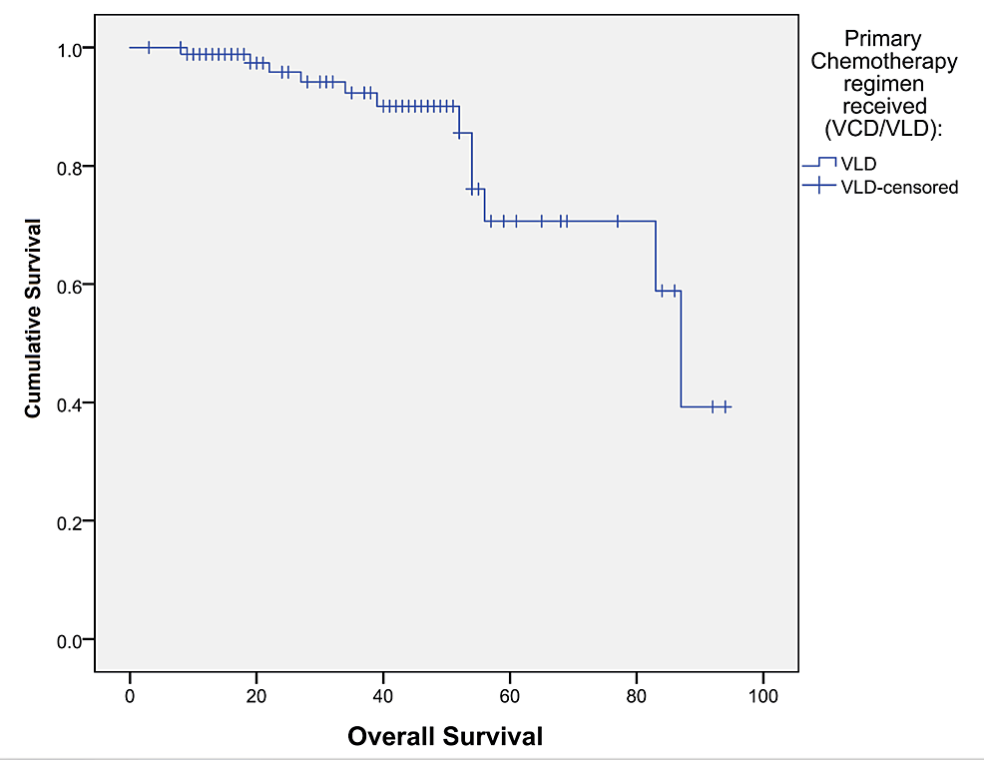
Kaplan-Meier survival graph of the group receiving VLD as the primary chemotherapy regimen The X-axis represents the overall survival in months, while the Y-axis represents the survival function represented as the cumulative survival. VCD: bortezomib/cyclophosphamide /dexamethasone; VLD: bortezomib/lenalidomide/dexamethasone

The mean survival time, which was calculated from the diagnosis, was 76.9 months (95%CI: 69.0-85.0) for the overall patient sample. The group that received the VCD regimen exhibited a mean survival time of 69.1 months (95%CI: 61.3-77.0), while those who received the VLD regimen had a mean survival time of 76.9 months (95%CI: 69.0-85.0) (Figure 1 and Figure 2). Therefore, there was no statistically significant difference between the mean survival times for the VCD and VLD groups. The PFS could not be calculated due to the low number of events of disease progression.

Following the completion of four cycles of therapy, 23.8% (n=20) of patients in the VCD group underwent complete remission, compared to 23.0% (n=20) in the VLD group (Table 2). The rate of partial remission was 33.3% (n=28) and 32.2% (n=28) in the VCD and VLD groups, respectively. Very few patients in either group experienced progressive disease, with 8.3% (n=7) in the VCD group and 3.4% (n=3) in the VLD group experiencing progressive disease. No patients underwent a stringent complete response in either group. The overall response rate stood at 69.0% (n=58) in the VCD group and 80.5% (n=70) in the VLD group, a difference that was not statistically significant on the Chi-square test (p=0.086) (Table 3).

**Table 2:** Comparison of response to treatment between the groups receiving VCD and VLD as the primary chemotherapy regime The table shows the number (n) and percentage (%) of patients in each response category for both the VCD group and VLD group. The response categories are as follows: CR: complete response, VGPR: very good partial response, PR: partial response, MR: minimal response, SD: stable disease and PD: progressive disease. VCD: bortezomib/cyclophosphamide /dexamethasone; VLD: bortezomib/lenalidomide/dexamethasone

Response	VCD, n (%)	VLD, n (%)
CR	20 (23.8)	20 (23.0)
VGPR	10 (11.9)	22 (25.3)
PR	28 (33.3)	28 (32.2)
MR	6 (7.1)	7 (8.0)
SD	13 (15.5)	7 (8.0)
PD	7 (8.3)	3 (3.4)

**Table 3:** Contingency table for the Chi-square test comparing response rates between the VCD and VLD groups The percentage of patients showing response with each treatment regimen has been represented as n (number of patients) and (%) (percentage of patients). A p-value of less than 0.05 has been taken as the cut-off for significance. Pearson Chi-Square (χ2) = 2.957, df = 1, p-value = 0.086

	VCD, n (%)	VLD, n (%)	Total, n (%)
Response	58 (69)	70 (80.5)	128 (74.9)
No Response	26 (31)	17 (19.5)	43 (25.1)
Total	84 (100)	87 (100)	171 (100)

The Cox regression revealed that none of the demographic variables had any bearing on the chances of survival. This included variables such as age (HR: 0.972, 95%CI: 0.938-1.007, p= 0.111), gender (HR: 0.464, 95%CI: 0.187-1.150, p= 0.097), hypertension (HR: 0.575, 95%CI: 0.268-1.234, p= 0.156), diabetes mellitus (HR: 1.772, 95 CI: 0.653-4.811, p= 0.262), and ischemic heart disease (HR: 0.502, 95%CI: 0.192-1.316, p= 0.161). Similarly, none of the baseline hematological parameters had any effect on survival, nor did radiation therapy (HR: 1.105, 95%CI: 0.394-3.098, p= 0.849) or the number of chemotherapy cycles received (HR: 0.929, 95%CI: 0.851-1.014, p= 0.097) have any effect (Table 4).

**Table 4:** Results of Cox regression For each variable, the hazard ratio (HR), corresponding 95% confidence intervals (CI) of the hazard ratio, and p-values have been reported in the form of: HR (lower limit of CI - upper limit of CI). A p-value of less than 0.05 was taken as the cut-off for statistical significance.

	Hazard Ratio (95% CI)	P - Value
Age (years)	0.972 (0.938 - 1.007)	0.111
Gender (Male, Reference: Female)	0.464 (0.187 - 1.150)	0.097
Hypertension	0.575 (0.268 - 1.234)	0.156
Diabetes mellitus	1.772 (0.653 - 4.811)	0.262
Ischemic heart disease	0.502 (0.192 - 1.316)	0.161
Radiation received	1.105 (0.394 - 3.098)	0.849
Number of chemotherapy cycles received	0.929 (0.851 - 1.014)	0.097

## Discussion

We conducted a retrospective cohort study to assess the outcomes of VLD and VCD therapy in NDMM patients. We found the mean survival time of NDMM patients undergoing VLD or VCD therapy to be 76.9 months (95%CI: 69.0-85.0), which was comparable to real-world experiences from high-income countries in Europe and the United States [12,13]. This finding demonstrates that these therapies can be applied as effectively in low- and middle-income countries (LMICs) as anywhere else in the world. This is also corroborated by similar studies on real-world experiences from other LMICs [14].

The overall response rate was found by our study to be 69.0% for VCD and 80.5% for VLD. This was lower as compared to other studies [12,13] as response rates for these two therapies have ranged anywhere from 88% all the way up to 100% [15,16]. In fact, a similar study conducted in Pakistan by Toor et al. found the response rate for VCD to be 88% and 89.4% in patients undergoing either VLD or VTD therapy [16]. The differences in response rate between our study and those conducted abroad could possibly be attributed to poor compliance owing to treatment costs, with bortezomib in particular being costly in the Pakistani market [17]. On the other hand, Toor et al. had a much smaller sample size than our study, which might explain these differences [16]. Additionally, our study could not detect any statistical significance in the difference in the overall response rate between VCD and VLD. This was in contrast to many other studies which found the response rate to be lower with VCD therapy compared to VLD therapy [12,13]. However, Toor et al., in another study conducted in the Pakistani setting, did not find the response rate of VCD to be inferior, although the study did not purely compare VCD to VLD, rather, it was compared to a sample of patients undergoing either VLD or VTD therapy [16].

Additionally, the present study was also unable to detect any statistically significant differences in the mean OS time between patients undergoing VCD therapy and those undergoing VLD therapy. In this context, the results in the literature are less consistent, with some studies similarly reporting no differences in the OS between VCD and VLD [13] while others reported a longer OS in patients undergoing VLD therapy [12].

One parameter frequently used to quantify and compare the effectiveness of VCD and VLD therapy in the literature was PFS. Although we attempted to calculate the PFS for our patient sample, due to the low number of events of relapse and relatively shorter person-time of observation, we were unable to calculate a meaningful median value for the PFS. Nevertheless, it is useful to examine what the literature says on the PFS for VCD and VLD, with studies having variously reported a longer PFS with VLD [12] or no statistical difference between VCD and VLD [13,14].

In our Cox regression, we were unable to find any associations between any of the demographic variables and clinic-hematological parameters and OS. Previous studies have identified many potential factors that could affect OS and/or the PFS, such as receiving a transplant [14].

Apart from the inability to calculate the mean PFS, another limitation of our study is the exclusion of any lab testing not done at the study institution as the results were not available in the hospital’s electronic records system. Additionally, any progression or death diagnosed at an institute outside the hospital’s network was unknown to us. Moreover, the unavailability of FLC ratios for most of our patients due to the prohibitive costs of testing made it difficult to evaluate stringent complete responses. This underscores the significance of cost-effectiveness for any diagnostic criteria in LMIC settings. Clinical guidelines should therefore take into consideration the applicability of their recommendations in such resource-limited settings. Other limitations of our study included the fact that it was a single-center study with a smaller sample size and the retrospective study design. On the other hand, the strengths of our study included the fact that it was the first such study, to our knowledge, conducted in a South Asian setting that compared the efficacies of these two regimens. Moreover, it demonstrated good outcomes in the LMIC setting, which were almost comparable to those from more resource-rich settings.

## Conclusions

The study did not identify any statistically significant distinction in the treatment outcomes between the VCD and VLD regimens among NDMM patients ineligible for transplantation. Nevertheless, the study highlights the positive outcomes observed with both treatments in this specific patient cohort. This implies that either regimen could be deemed suitable as a treatment option for patients in LMICs. Since both regimens demonstrate comparable effectiveness, assessing the cost-effectiveness of these regimens is crucial. Future research should also explore the economic aspects of the two treatment options.
